# Healthier Factories

**Published:** 1920-10-16

**Authors:** 


### Healthier Factories.

### THE EFFECT OF SHORTER HOURS.

The Chief Inspector of Factories, in his report for 1919, gives some valuable information on the effect of shorter hours on health and production; and he anticipates a time when the overworked man or woman will rarely be found throughout industry. The standard of safety and sanitation which declined during the war has made great advance, in many cases far beyond the pre-war level. The Chief Inspector says the shortening of the working hours has had a beneficial effect on the operatives, perhaps more so than any other recent improvement in industrial conditions. Perhaps the classes of workers upon whom shorter hours will have the most beneficial effect will be the growing girl or boy, on whom long periods of employment told hardly, and the worker possessing some vision and aspiration towards a fuller life.

Better timekeeping had resulted from the discontinuing of work before breakfast, and there were less frequent absences owing to sickness. Less fatigue and overstrain is found in factories, and one inspector reports that, although more men are employed in engineering and allied trades, the accident list has not increased, and it is claimed that fatigue is non-existent. The workers are using their leisure in their allotments and gardens. Social clubs are getting better and keener attendance at technical and educational classes, and successful work schools are carried on in many factories. Far more, however, needs to be done in this way and in the promotion of recreations, especially for girls.

The effect of shorter hours on production is interesting. When the production depends almost entirely on the speed of machinery the output is said to be reduced in a proportion nearly, if not fully, corresponding to the reduction in hours. In other machine operations which call for constant alertness on the part of the operator output has not suffered to this extent, and, in exceptional cases, had scarcely been affected at all. In a third class of process, where output was largely or entirely dependent upon the exertion of the worker, there was frequently no loss in production, and in some cases even an increase.

